# Psychiatrist-led treatment of hepatitis C (HCV) at an opioid agonist treatment (OAT) clinic in Stockholm - enhancing the HCV treatment care cascade

**DOI:** 10.1192/j.eurpsy.2022.468

**Published:** 2022-09-01

**Authors:** M. Kåberg, P.-E. Klasa, T. Nordin

**Affiliations:** 1 Karolinska Institutet, Department Of Medicine Huddinge, Division Of Infectious Diseases, At Karolinska University Hospital Huddinge, Stockholm, Sweden; 2 Stockholm Center for Dependency Disorders, The Stockholm Needle Exchange, Stockholm, Sweden; 3 Prima Maria, Oat Clinic, Stockholm, Sweden

**Keywords:** Cascade of care, Opioid agonist treatment, People who inject drugs, Hepatitis C

## Abstract

**Introduction:**

People who inject drugs (PWID) and opioid agonist treatment (OAT) patients have an increased hepatitis C (HCV) prevalence. Studies among these populations show promising HCV treatment results, which is essential to reach the WHO goal of eliminating HCV as a major public health threat by 2030.

**Objectives:**

To introduce psychiatrist-led HCV treatment at an OAT clinic and to investigate HCV treatment results, i.e. sustained virological response at 12 weeks post treatment (SVR12) and numbers of reinfections.

**Methods:**

Prima Maria OAT clinic in Stockholm, provides OAT for 450 patients. The majority have a history of injection drug use. Baseline HCV prevalence (January 2018) was retrospectively investigated through medical charts. In January 2018, psychiatrist-led HCV treatment (with consultation support from infectious diseases specialists) was introduced at the clinic. Prospective treatment results, numbers of reinfections and incidence rates between January 2018 and April 2021 were further investigated.

**Results:**

Baseline data (n=418), showed that 46% were not tested for HCV. Of those tested (n=225), 64% had a chronic HCV infection. By January 2021, 104 HCV treatments were initiated. 97/97 (100%) were HCV RNA negative at end-of-treatment. 78/88 (89%) reached SVR12. Overall, 2 reinfections were noted after SVR12 corresponding to a reinfection rate of 3.5/100 PY. Numbers of HCV treatment did not decrease during the COVID-19 pandemic.

**Figure:**
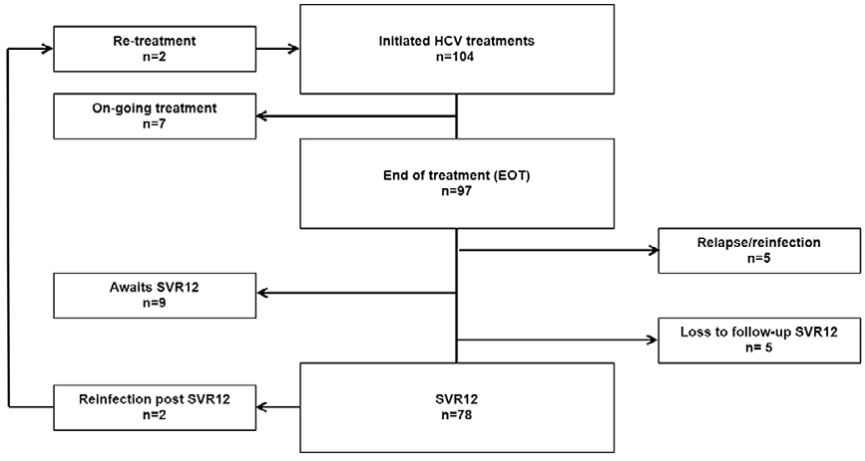

**Conclusions:**

To enhance the HCV treatment cascade, targeted HCV diagnosis efforts are needed. Bringing HCV treatment to OAT clinics enhance the HCV care cascade. HCV treatment education for psychiatrists/addiction specialists makes HCV treatment more sustainable, as specifically noted during the COVID-19 pandemic.

**Disclosure:**

This study was partly funded by Gilead Nordic Fellowship 2020. The funders had no role in study design, data collection and analysis, decision to publish or preparation of the poster/manuscript.

